# Evaluation of Porcine Collagen Membranes Used with Guided Bone Regeneration for Critical Defects: A Histological, Histomorphometric, Immunohistochemical, and Inflammatory Profile Analysis

**DOI:** 10.1055/s-0043-1777045

**Published:** 2024-01-23

**Authors:** Edith Umasi Ramos, Miguel Nino Chávez Leandro, Jesús Omar Cárdenas Criales, Marisol Rossana Ortega Buitron, Edgar Simón Verástegui, Wilbert Manzanedo Carbajal, Ronald Christian Solís Adrianzén, Anibal Eleuterio Espinoza Grijalva, Antonio Alberto Ballarte Baylon, Ana Paula Farnezi Bassi

**Affiliations:** 1 Diagnosis and Surgery Department, School of Dentistry, São Paulo State University (UNESP), Arçatuba, São Paulo, Brazil.; 2 School of Dentistry, Universidad Nacional Hermilio Valdizan, Huánuco, Perú.

**Keywords:** bone regeneration, guided tissue regeneration, collagen, inflammation

## Abstract

**Objective** The objective of this study was to compare the effectiveness of two porcine collagen membranes of different origin used for guided bone regeneration procedures.

**Materials and Methods** Resorbable collagen membrane from porcine dermis (Bio-Gide, Geistlich Pharma AG, Wolhusen, Switzerland) and resorbable collagen membrane from porcine pericardium (Jason, Institut Straumann AG, Peter Merian-Weg, Switzerland) were evaluated; histological, histometric, immunohistochemical, and inflammatory profile analyses were performed. The study was carried out on critical defects created in the calvaria of 72 rats ( *Rattus norvegicus albinus* , Wistar variety) divided into three groups: coagulum group (Co), porcine pericardium group (JS), and porcine collagen group (BG). The defects were filled with clot, over which the membranes were placed. The animals were euthanized 7, 15, 30, and 60 days after surgery.

**Statistical Analysis** The Shapiro–Wilk test was used to assess data distribution. Analysis of variance (ANOVA) and the Bonferroni multiple comparison test were used to compare the differences across the mean values of the variables. Nonparametric tests, Mann–Whitney and Wilcoxon W, were used for the quantitative analysis of the inflammatory profile. A significance level of 5% ( *p* < 0.05) was adopted with a confidence interval of 95%. SPSS software version 2.0 was used.

**Results** A total of 1,008 analyses were performed on 288 histological slides. It was noted that both types of collagen membranes used in this study were effective for the guided bone regeneration procedure, with a greater proportion and thickness of bone formation among recipients of the BG (735 points, *p* = 0.021). This membrane also had greater permeability (62.25). The animals in the JS group, which received the porcine pericardial membrane, showed early and accelerated bone formation from early bone tissue, milder osteopontin and osteocalcin levels, and greater inflammatory reaction (86.4).

**Conclusion** The collagen membrane from porcine dermis demonstrated a more orderly and physiological repair process, while the porcine pericardial membrane presented a more accelerated repair process that did not remain constant over time.

## Introduction

The most important determinants of success in guided bone regeneration (GBR) procedures are the membranes; accordingly, deep knowledge of the properties of these materials is essential. These membranes are classified as absorbable and nonabsorbable. 1 Among the absorbable membranes, collagen membranes (CMs) are widely used and have shown good results for over 30 years, without being free of complications. 2

The CMs currently used in GBR are bovine and porcine derivatives, obtained from various structures such as the bovine Achilles tendon and internal organs such as the peritoneum, pericardium, and porcine dermal matrix. 3 4 There are so far 28 types of collagen 5 ; CMs for GBR are made up of type I and III collagen. 4 Collagen provides properties such as elasticity and mechanical strength. 6

For bone formation to occur, however, ample blood support and mechanical support are imperative, because osteoblasts synthesize bone matrix proteins close to the vessels. 7 CMs have a hemostatic function, in addition to capabilities to allow for wound stability and permeability to facilitate the transfer of nutrients. 6 These membranes must have certain fundamental characteristics, such as biocompatibility, stability, and adequate resorption time. 8 Grafts of porcine origin also have a similar degree of compatibility with grafts of human origin. 9

In GBR, success depends on the rate of resorption as well as the membrane's effectiveness as a barrier 8 to remain long enough for repair to occur. 4 A wide variety of CMs are on the market, such as Bio-Gide (Geistlich Pharma AG, Wolhusen, Switzerland), which has high density and low porosity and is one of the most studied and utilized membranes in regeneration processes. 10 It comprises two layers, which together prevent invagination of soft tissue into the defect. This membrane ensures efficient bone formation and space maintenance; it also prevents particle migration and aids clot formation, thus obtaining more bone of superior quality. 11 Another CM recently introduced in the market, Jason (Institut Straumann AG, Peter Merian-Weg, Switzerland), is composed of collagen extracted from porcine pericardium and formed by type I and III collagen, with a higher proportion of type I collagen, a degradation time of 12 to 28 weeks, reduced thickness, high resistance to rupture, and easy moldability and adaptability to bone surface. 4

In GBR, the use of CMs has the disadvantage of rapid biodegradation. This represents one of the most challenging aspects for space maintenance, as the loss of bone volume arising from compromised mechanical support can lead to collapse in bone defects, 3 ultimately resulting in failure of the bone structure regeneration. 12 Another consideration is the ample blood support related to the porosity of the membranes, which favors CMs. However, the greater the porosity, the faster the resorption. Although the described properties of porcine CMs, specifically porcine pericardium membrane (Jason, Institut Straumann AG), are promising, it is difficult to know the exact period that these membranes remain. Only a few clinical studies on this topic exist, and the lack of animal model studies similar to this one makes this research especially relevant.

In this study, we evaluated porcine CMs in an animal study through histological, histomorphometric, and inflammatory profile analyses. Our objective was to evaluate and compare the GBR process, as well as to assess the degradation patterns of porcine CMs of dermal and pericardial origin.

## Material and Methods

### Materials

Resorbable collagen membranes from porcine dermis (Bio-Gide, Geistlich Pharma AG) and porcine pericardium (Jason, Institut Straumann AG) were used in the study.

### Experimental Model

The study was approved by the Animal Experimentation Ethics Committee of School of Dentistry, Araçatuba, UNESP (CEUA), Process n= 00372 2018. A total of 72 rats ( *Rattus norvegicus albinus* , Wistar variety) were selected for the study. To determine this sample number of animals, the sample was calculated as *n* = 6 for each time of euthanasia. The power test was performed by statistically calculating the power of the test on the website http:/www.biomath.info/power/prt.htm based on previous results in the study by Ramires et al. 13 These were males of 3 to 4 months weighing 200 to 300 g, divided into three groups of 24 rats each. The groups were the following: group co, coagulum group (defect filled with clot without use of membrane); group JS, porcine pericardium group (defect filled with clot and porcine pericardium membrane [Jason, Institut Straumann AG] placed over the defect); and group BG, porcine collagen group (defect filled with clot and porcine collagen membrane of dermal origin [Bio-Gide, Geistlich Pharma AG] placed over the defect). The animals were kept in a vivarium, fed with balanced solid rations (Nuvilab, Curitiba, PR, Brazil) containing 1.4% Ca and 0.8% P and water ad libitum throughout the experiment, except for the period of 12 hours prior to the surgery. Each animal was identified with a number on the ear. Each group was divided into four subgroups ( *n* = 6 per group), according to the times of euthanasia: 7, 15, 30, and 60 days after surgery. The calvaria used in the study followed a standard model that allowed for the assessment of bone formation capacity in the defect. 14

### Surgical Protocol

**Fig. 1:**
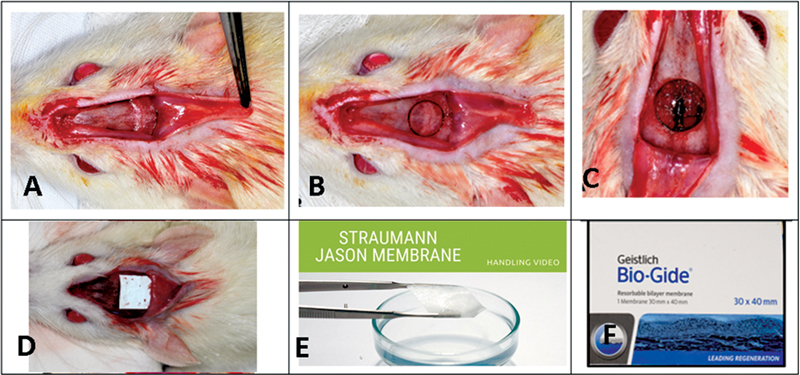
Surgical protocol. ( **A** ) Surgical access in the calvaria of rats. ( **B** ) Bone defect of 7 mm in the center of the calvarias. ( **C** ) Cortex removed from the critical defect. ( **D** ) Collagen membrane on surgical defect. ( **E** ) Membrane from porcine pericardium. ( **F** ) Membrane from porcine dermis.

Sedation was induced by intramuscular administration with ketamine hydrochloride (Francotar-Virbac do Brasil Ltda, São Paulo, Brazil), 0.07 mL per 100 g of weight, associated with xylazine (Rompun, Bayer AS, Saúde Animal, São Paulo, Brazil), 0.03 mL per 100 g of weight. Trichotomy was performed on the calvaria; antisepsis was performed with polyvinylpyrrolidone iodine (PVPI 10%, Riodeine Ind. Farm., Rioquímica Ltda). The incision area was infiltrated with 0.3 mL/kg 2% mepivacaine with epinephrine (1:100,000, Mepiadre 100, FDL LTDA, Rio de Janeiro, Brazil); then a **V** -shaped incision was made in the anteroposterior direction, with total detachment of the flap, maintaining the integrity of the dura mater. Subsequently, with a 7-mm internal diameter trephine drill attached to a 20:1 low-rotation contra-angle under irrigation with 0.9% sodium chloride solution, a critical surgical defect measuring 7 mm in diameter was made in the central portion of the calvaria 15 ( Fig. 1A–C ). All defects were filled with clot; no membrane was placed over the defect in the clot group, and CMs were used in the other two groups ( Fig. 1D ). The surgeon was unaware of which membrane was placed; the tissues were repositioned and sutured with monofilament thread (Nylon 5.0, Mononylon, Ethicon, Johnson Prod., São José dos Campos, Brazil). Penicillin G-benzathine (Pentabiotico Veterinário, Fort Dodge Saúde Animal Ltda., Campinas, SP, Brazil) and sodium dipyrone were administered intramuscularly in 0.2-mL single doses. The animals were kept in cages and euthanized with an excessive dose of anesthetic in accordance with the euthanasia times. A total of 12 study groups were obtained: Co (7, 15, 30, and 60 days); JS (7, 15, 30, and 60 days); and BG (7, 15, 30, and 60 days).

### Material Collection and Histological Preparation

The calvaria of the removed rats were fixed in a 10% formaldehyde solution (Analytical Reagents, Dinâmica Odonto-Hospitalar Ltda, Catanduva, SP, Brazil) for 48 hours, washed in running water for 24 hours, decalcified in 20% EDTA (ethylene diamine tetraacetic acid, Merck) for 5 weeks, dehydrated in a sequence of alcohols, and cleared. Subsequently, the calvaria were cut in half longitudinally, separating the bone defects. The pieces obtained were embedded in paraffin, cut to 6-μm thickness, and mounted on slides. The slides were stained in hematoxylin and eosin (H&E).

### Histological Analysis

An experienced investigator performed the analyses, using an optical microscope (Leica DMLB, Heerbrugg, Switzerland) coupled to an image capture camera (Leica DC300F, Leica Microsystems Ltd., Heerbrugg, Switzerland) and connected to a Pentium III microcomputer with Image Lab 2000 image analyzer software (Image Processing and Analysis Software, Ontario, Canada). Images were recorded in JPEG files, and analyzed at 6X, 25X, and 40X magnifications. Presence of newly formed bone tissue, membrane, connective tissue, inflammation, and degree of membrane resistance (collapse) was evaluated.

### Histometric Analysis

An optical microscope (Leica DMLB, Heerbrugg, Switzerland) coupled to an image capture camera (Leica DC300F, Leica Microsystems Ltd, Heerbrugg, Switzerland) was used to obtain the images, which were stored as JPEG files and analyzed in the ImageJ program (National Institutes of Health, Bethesda, MD, United States). Quantitative analysis was carried out in eight captures with 12.5X magnification. In the plug-ins tab, the Grid tool was used to create a tab containing 423 crosses. The area of new bone formation was counted in all fields to yield a total of newly formed bone tissue for each animal.

### Immunohistochemical Analysis

The immunoperoxidase detection method was applied, using secondary biotinylated antigoat antibody (Jackson ImmunoResearch Laboratories). The marker signal amplifier was avidin/biotin (Elite Kit, Vector Laboratories) with diaminobenzidine (Dako) as chromogen, using osteopontin and osteocalcin (Santa Cruz Biotechnology) to label cells of osteoblastic lineage and nonmineralized and mineralized extracellular matrices at different times. OP is present at the beginning of mineralization, in addition to being a marker of reversal lines in bone tissue; osteocalcin is present in the final stage of mineralization, characterizing a more mature tissue. For the antibodies, the expression of these proteins was evaluated semiquantitatively by attributing different scores according to the number of immunostained cells in the bone repair process in different areas of the defects.

### Inflammatory Profile Analysis

For this analysis, ImageJ Software (National Institutes of Health, United States) was used with the aid of the plug-in tool Cell Counter. Selected fields were photographed at 100X magnification with a microscope (Leica DMLB) coupled to an image capture camera (Leica DC300F, Leica Microsystems Ltd, Heerbrugg, Switzerland). Counts of the numbers of inflammatory cells (polymorphonuclear, mononuclear, and osteoclasts), and the number of blood vessels in all slides of the JS and BG groups were performed at 7 and 15 days. 16 In addition, the intensity of the inflammatory process was evaluated by analyzing the number of inflammatory cells present in each cut. 17 The scores were the following: score 1 = absent (fewer than three per field); score 2 = slight (fewer than 10); score 3 = moderate (10–25); and score 4 = intense (more than 25). Results are expressed as average for later statistical analysis.

For the histological and histometric analyses, they were carried out by a single evaluator previously calibrated for this purpose, as well as for the immunohistochemical analysis, another evaluator, and the inflammatory profile analysis, also by another evaluator, both trained and calibrated prior to the evaluation process.

### Statistical Analysis

Quantitative variables were expressed as mean and standard deviation. The Shapiro–Wilk test was used to assess data distribution. Analysis of variance (ANOVA) and the Bonferroni multiple comparison test were used to compare the differences across the mean values of the variables. Pearson's correlation test, in addition to two nonparametric tests, Mann–Whitney and Wilcoxon *W* , were used for quantitative analysis of the inflammatory profile. Student's *t* -test was performed to assess equality of means. A significance level of 5% ( *p* < 0.05) was adopted with a confidence interval of 95%. 17 SPSS software version 2.0 was used.

## Results

### Seven Days

**Fig. 2:**
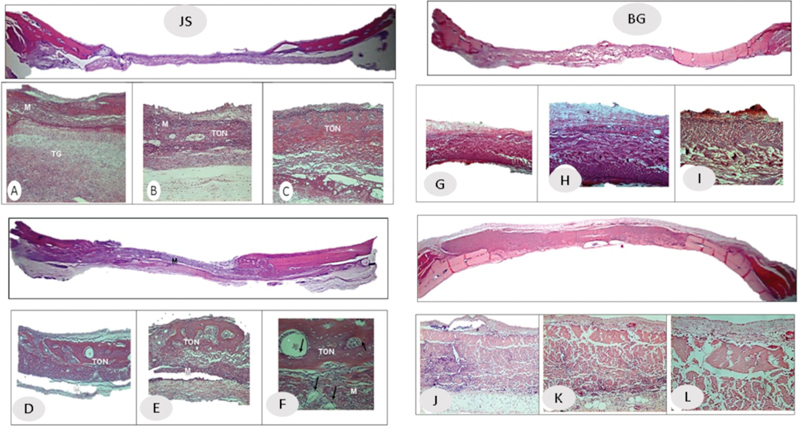
Histological analysis in the porcine pericardium group (JS) at ( **A–C** ) 7 days after surgery and ( **D–F** ) 15 days after surgery. Histological analysis in the porcine collagen group (BG) at ( **G–I** ) 7 days and ( **J–L** ) 15 days.

*Histological analysis:* In the panoramic views of the JS and BG groups, the membrane could be seen throughout the entire length of the defect ( Fig. 2 ); the Co group defect was filled by granulation tissue. At 6.3X and 12.5X magnifications, granulation tissue and several membrane layers were observed in the JS group ( Fig. 2 **A, B** ), while in the BG group, highly vascularized granulation tissue was observed without inflammatory infiltrate. With 25X magnification, bone tissue was observed inside the membrane ( Fig. 2G **; BG** ). In some cases, a collapsed membrane was observed in the JS group and in the BG group, the beginning of a newly formed bone was observed close to the stumps. In the Co group, little extracellular matrix in the central region of the wound was observed.

**Fig. 3:**
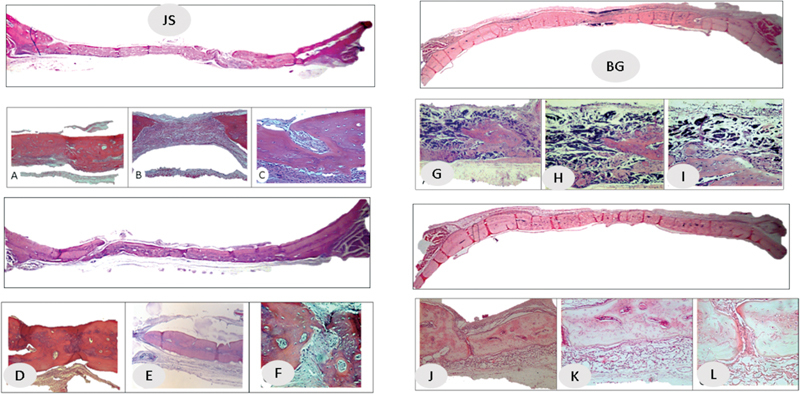
Histological analysis in the porcine pericardium group (JS) at ( **A–C** ) 30 days and ( **D–F** ) 60 days. Histological analysis in the porcine collagen group (BG) at ( **G,H** ) 30 days and ( **J–L** ) 60 days.

**Fig. 4:**
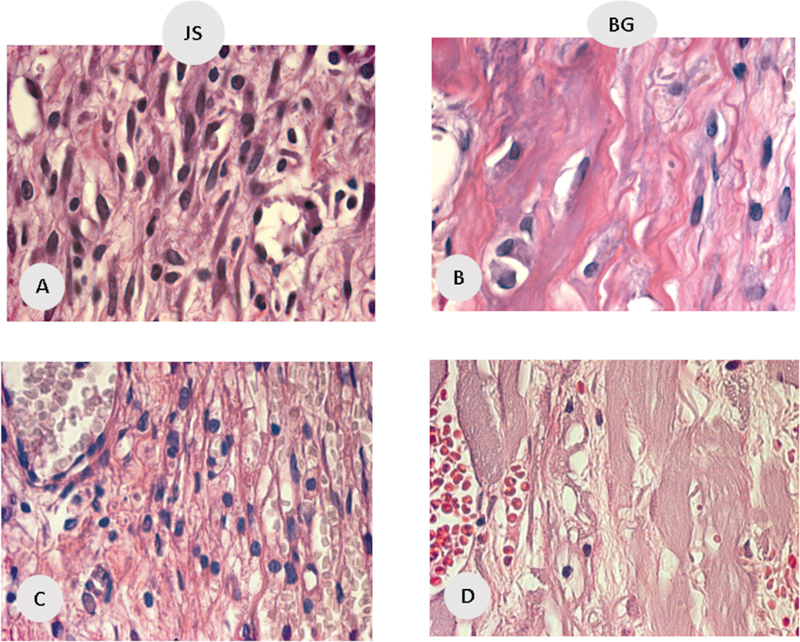
Inflammatory profile. ( **A** ) Blood vessels in the porcine pericardium group (JS) at 7 days after surgery; ( **B** ) blood vessels in JS at 15 days after surgery. ( **C** ) Inflammatory cells in the porcine collagen group (BG) at 7 days after surgery. ( **D** ) Inflammatory cells vessels in BG at 15 days after surgery.

*Histometric analysis:* The JS group averaged 199.2 points, the BG group averaged 196.2 points, and the Co group averaged 115.6 points ( Table 1 ). Comparing the JS group with the BG group, *p* = 1.0; between groups BG and Co, *p* = 1.06, without significant differences between the groups. Between groups JS and Co ( *p* = 0.08), there was a statistically significant difference.

**Table 1:** Proportion of neoformed bone in the porcine pericardium (JS) and porcine dermis (BG) groups

Groups	7 d	SD	15 d	SD	30 d	SD	60 d	SD
BG	196.2	22.8	343.6	64	888.2	72.7	1,423	64.7
JS	199.2	29.2	494.2	19.8	979.8	139.8	1,151.6	77.7
*p* < 0.05	1.00		0.01		0.052		0.00	

Abbreviations: BG, porcine dermis membrane group; JS, porcine pericardium membrane group; SD, standard deviation, 95% confidence interval.

**Fig. 5:**
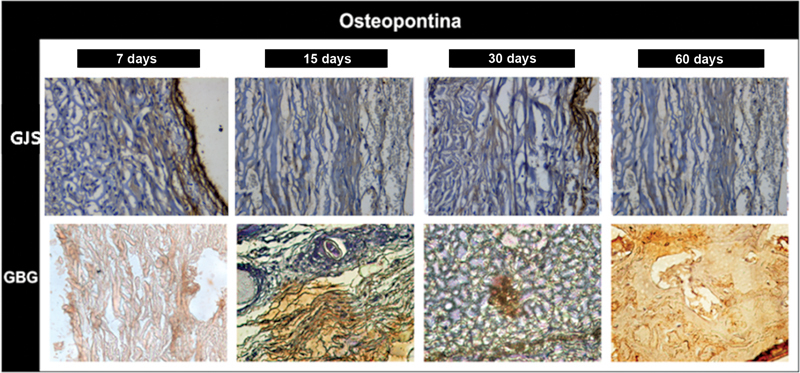
Immunohistochemical analysis. Osteocalcin in the porcine pericardium group (JS) at 7, 15, 30, and 60 days after surgery and in the porcine collagen group (BG) at 7, 15, 30, and 60 days after surgery. GSJ, porcine pericardium group; GBG, porcine collagen group.

**Fig. 6:**
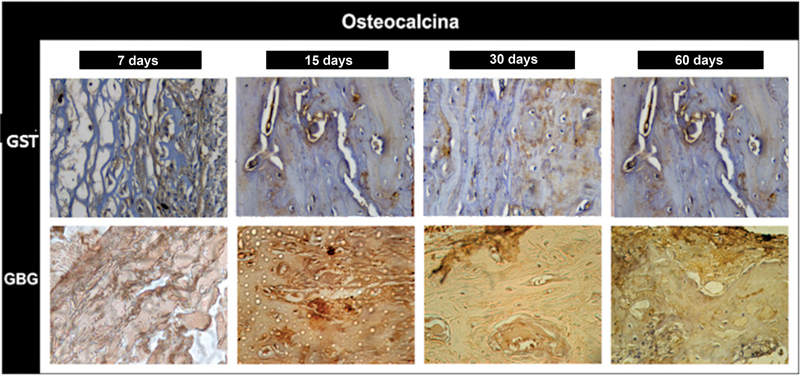
Immunohistochemical analysis. Osteocalcin in the porcine pericardium group (JS) at 7, 15, 30, and 60 days after surgery and in the porcine collagen group (BG) at 7, 15, 30, and 60 days after surgery. GSJ, porcine pericardium group; GBG, porcine collagen group.

*Immunohistochemical analysis:* osteopontin in group JS: discrete immunostaining in the extracellular matrix (score 1; Fig. 5 and Table 2 ). Osteopontin in group BG: moderate immunostaining (score 2). Osteocalcin in group JS: moderate immunostaining; areas of precipitated minerals on the newly formed bone matrix (score 2). Osteocalcin in group BG: moderate immunostaining (score 2; Fig. 6 and Table 2 ), showing the beginning of the bone formation process.

**Table 2:** Scores of immunohistochemical analysis of the osteocalcin and osteopontin

Groups	Osteocalcin	Osteopontin
Group ST 7 d	++	+
Group BG 7 d	++	++
Group ST 15 d	+++	++
Group BG 15 d	++	++
Group ST 30 d	+++	+
Group BG 30 d	+++	+
Group ST 60 d	++	+
Group BG 60 d	+++	+

Abbreviations: Group ST, porcine pericardium membrane group; group BG, porcine dermis membrane group.

*Inflammatory profile analysis:* The number of inflammatory cells in group JS was 86.4, while in group BG, it was 64.8 ( *p* = 0.81). The number of blood vessels in group JS was 42.2, while in group BG, it was 68.5 ( *p* = 0.21). There was no statistically significant difference in the correlation between the number of inflammatory cells and new bone formation, as well as in the number of blood vessels and new bone formation.

### Fifteen Days

*Histological analysis:* The panoramic view shows the integrated membrane covering the entire defect in the JS and BG groups ( Fig. 2 ). Magnification of 6.3X to 25X shows islands of bone in the center of the defect, presence of bone throughout, and membrane and highly vascularized tissue in the JS group. In group BG, areas of newly formed bone and highly vascularized tissue from the stump were observed ( Fig. 2G–I ). In group Co, the bone defect was filled with loose connective tissue. At higher magnification, new bone formation was observed in the stumps of the defect.

*Histometric analysis:* Group JS averaged 494.2 points, group BG averaged 343.6 points, and group Co averaged 122.4 points ( Table 1 ). Comparing group JS with group BG, *p* = 0.01; between groups BG and Co, *p* = 0.00; and group JS and Co, *p* = 0.0, all with statistically significant differences.

*Immunohistochemical analysis:* Osteopontin in group JS: moderate immunostaining in the extracellular matrix, and cells of osteoblastic lineage (score 2; Fig. 5 ). Osteopontin in group BG: moderate immunostaining in the extracellular matrix (score 2). Osteocalcin in group JS: intense immunostaining in areas of mineralization (score 3; Fig. 6 ). Osteocalcin in group BG: moderate immunostaining (score 2).

*Inflammatory profile analysis:* The number of inflammatory cells in group JS was 80.5, while in group BG, it was 48.75 ( *p* = 0.38), with no statistically significant difference in the correlation between the number of inflammatory cells and new bone formation. The mean number of blood vessels in group JS was 33.2; in group BG, it was 62.25 ( *p* = 0.00), with a statistically significant difference in the correlation between the number of blood vessels and new bone formation.

### Thirty Days

*Histological analysis:* In the panoramic view, the entire defect is apparently closed in both the JS and BG groups ( Fig. 3 ). At 6.3X and 12.5X magnification, the centers of the defect appear partially closed ( Fig. 3 A , B **(JS); G, H (BG)** .). Dense connective tissue and the presence of membrane fragments were observed in groups JS and BG. In group Co, a smaller defect was observed, the center of which was filled with connective tissue. At higher magnification, areas of newly formed bone tissue were observed close to the stump, with loose connective tissue in the rest of the defect.

*Histometric analysis:* Group JS averaged 979.8 points, group BG averaged 888.2 points, and group Co averaged 258.4 points ( Table 1 ). Comparing groups JS and BG, *p* = 0.052, there was no statistically significant difference. Between groups BG and Co ( *p* = 0.00) and between groups JS and Co ( *p* = 0.00), there was a significant difference.

*Immunohistochemical analysis:* Osteopontin in group JS: discrete immunostaining in the extracellular matrix, and cells of osteoblastic lineage (score 1; Fig. 5 ). Osteopontin in group BG: discrete immunostaining in the extracellular matrix and moderate staining in the stumps (score 1). Osteocalcin in group JS: intense immunostaining in the extracellular matrix and in some cells (score 3); osteocalcin in group BG: intense immunostaining (score 3; Fig. 6 ).

### Sixty Days

*Histological analysis:* In the panoramic view of group JS, the defect is filled with bone tissue ( Fig. 3 ). At 6.3X magnification, some slides show the center completely to be filled or with small areas of connective tissue ( Fig. 4D–F ). At 12.5X to 25X, membrane remnants are still observed. In the panoramic view of group BG ( Fig. 4J–L ), the bone defect is completely regenerated. At higher magnification, the center and the stump show the presence of newly formed bone tissue, connective tissue, and osteoid tissue, along with some membrane remnants. In group Co, a smaller defect is observed, without signs of new bone formation in the center and with the presence of immature connective tissue.

*Histometric analysis:* Group JS averaged 1151.6 points, group BG averaged 1,423.3 points, and group Co averaged 380 points ( Table 1 ). Comparing group JS with group BG, *p* = 0.001; between groups BG and Co, *p* = 0.00; and between groups JS and Co, *p* = 0.00, all with significant difference.

*Immunohistochemical analysis:* Osteopontin in group JS: discreet immunostaining in the nonmineralized extracellular matrix (score 1; Fig. 5 ). Osteopontin in group BG: mild immunostaining in the extracellular matrix, and more organized bone trabeculae (score 3). Osteocalcin in group JS: mineralized extracellular matrix with regions of osteocalcin precipitation, mineralization of the newly formed tissue, and presence of cells of osteoblastic lineage (score 2). Osteocalcin in group BG: light immunostaining, already marking a process of bone maturation (score 1; Fig. 6 ).

## Discussion

Since the general objective of the study was to evaluate bone formation, it is essential to consider two aspects: mechanical support and blood supply. 7 In this respect, we know that the main collagen component of the two membranes under investigation have the disadvantage of reduced stability. 18 We observed in this study that the porcine pericardium membrane had lower mechanical resistance compared with the porcine dermal membrane, with collapse being observed; also, the histometric result at 60 days, *p* = 0.001, showed a significant difference. The result of our study is similar to what was observed in the study by Susanto et al 19 where the membrane they used had ideal porosity for guided tissue regeneration but lower mechanical resistance.

Another factor that affects stability is degradation time. 20 Degradation time of the porcine pericardial membrane ranged from 12 to 28 weeks, 21 while that of the porcine dermal membrane was 16 to 24 weeks as specified by the manufacturers. This indicates that the degradation times are similar for the two membranes. Membranes require 6 months for the bone tissue regeneration process and 4 weeks for periodontal regeneration. 22 In this study, with histological analysis at 15 days, the membranes of both groups JS and BG were superficially integrated. Considering that the repair process in rats is four times faster than that of humans, 23 15 days in this study would equate to 60 days in humans. At 30 days (equivalent to 4 months in humans), none of the membranes were intact, which means that between 60 and 120 days these membranes are no longer barriers in the GBR process.

The second aspect, blood supply, is an important factor in providing nutrients and facilitating bone formation 20 24 as was also observed in the study by Linawati et al 25 that when there is a greater blood supply, the wound healing process is accelerated. Porcine pericardial membrane is half as thick and three times less dense than porcine collagen membrane of dermal origin, 18 so the vascular supply should be greater. This study demonstrated that the quantity of blood vessels was smaller in group JS at 7 and 15 days, and the difference was significant at 15 days. This result contradicts the high porosity described in the product catalog and in the study by Ortolani et al. 21 This fact could explain why the histometric analysis of group JS at 7, 15, and 30 days was superior to that of group BG.

In the study by Rothamel et al, 4 the porcine pericardial membrane allowed for a greater level of osteoblast cell proliferation after 7 days than the porcine collagen membrane of dermal origin; this would suggest greater bone formation, a finding also observed in this study. A greater proportion of bone formation was observed in group JS, the porcine pericardial membrane group, in addition to a greater number of inflammatory cells at 15 days. These factors interfere in the amount of newly formed bone at 30 and 60 days, already well discussed by Patino et al. 7 One hypothesis is that the degradation of the porcine pericardial membrane causes an inflammatory reaction greater than that of the porcine dermal membrane, crucial events affecting the final volume of newly formed bone tissue.

Regarding bone formation at the alveolar level, the study by Hassumi et al 26 notes that the process takes 28 days, which for humans represents 64 days. This bone regeneration has different phases, including clot formation, followed by the proliferation of immune cells (i.e., inflammatory cells). When collagen-based material is used, we mainly observe macrophages, cytokines such as interleukin-10 (IL-10). In our study with porcine pericardium membrane, an increased number of inflammatory cells was observed up to 15 days, reinforcing what was observed through histometric analysis, that is, rapid bone formation in the initial phase.

In the period of 7 and 15 days, the immunostaining for osteopontin is discrete and the osteocalcin is higher in group JS, a fact that would indicate formation of early bone tissue in the initial intervals (7 and 15 days). This does not remain consistent to the point of continuing at an accelerated pace in the 30- and 60-day periods. With the porcine collagen membrane group (BG), a stable structure was observed for up to 15 days, demonstrating a more orderly and physiological repair process; at 7 and 15 days, it presented higher immunostaining for osteopontin, and at 30 and 60 days the presence of osteocalcin was more significant. Another fact observed in the 60-day period was the existence of a moderate immunostaining for osteopontin in group JS; this may suggest that the remodeling process is beginning in this period, since the presence of tissue reversion line close to the stumps was observed, as well as bone at different levels of neoformation.

A study by Merli et al compared new bone formation for horizontal rehabilitation in humans using two combinations: porcine collagen membrane associated with inorganic bovine bone and porcine pericardial membrane associated with synthetic bone. The result indicated no significant differences between these two groups, with little difference in peri-implant bone loss, with better effects in the porcine collagen membrane group. 27 Our study corroborates the findings of Merli et al, reporting a similar bone neoformation pattern in the initial periods up to 30 days.

It was possible to verify that the porcine pericardial membrane has a shorter period to complete new bone formation, because after 15 days, it presents higher levels of mineralization than the porcine collagen membrane of dermal origin. However, in the 60-day period, the porcine collagen membrane group (BG) reached a higher rate of new bone formation than the porcine pericardium membrane group (JS). The porcine pericardium group (JS) allows superior bone formation in the initial periods compared with the membrane group BG.

With these data, we can suggest that the JS membrane would prove to be effective in performing bone regeneration in those patients with nutritional deficiencies or with compromised immunity or in the cases in which the bone defect is small and can be easily regenerated in a short period of time

## Conclusion

We can conclude that (1) porcine pericardium membrane could be useful for bone regeneration procedures that are less complex or that require a faster process and (2) porcine dermal membrane may be appropriate for complex, slower procedures or for patients with health conditions that do not guarantee good blood support. Both were deemed effective for the regeneration process in critical defects in the calvaria of rats.
